# Legal Considerations of COVID-19 Patients’ Disposition in Emergency Department; Report of 10 Cases

**Published:** 2020-06-27

**Authors:** Dorsa Najari, Alireza Zali, Fares Najari, David Soroosh

**Affiliations:** 1 Functional Neurosurgery Research Center, Shohada Tajrish Neurosurgical Center of Excellence, Shahid Beheshti University of Medical Sciences, Tehran, Iran.; 2 Department of Legal Medicine, Sabzevar University of Medical Sciences, Sabzevar, Iran.

**Keywords:** COVID-19, severe acute respiratory syndrome coronavirus 2, Legal Considerations, Forensic Medicine, Legal Medicine

## Abstract

COVID-19 pandemic is a challenge in the current era. The spread of this viral infection began in Wuhan City in China, and Iran was also one of the countries struggling with it. Considering the nature of this virus and the current pandemic, it is essential that the healthcare system authorities issue a clear and firm law on treating people infected with COVID-19 to prevent the consequences affecting the professional life of physicians and healthcare staff. The current study aimed at evaluating the legal consequences of COVID-19 cases in emergency department (ED). This case series reported 10 patients that filed complaints against medical staff for problems that occurred on arrival, during the hospital stay or discharge in Shohada-ye-Tajrish and Shahid Modarres educational Hospitals, Tehran, Iran. Consultation with forensic medicine department was requested for all patients and the final decision for each case was reported under the title legal considerations.

## Introduction

COVID-19 is a newly emerging viral disease, whose spread began in Wuhan City, China, in December 2019 and rapidly spread worldwide (1-4). Iran was one of the affected countries that experienced extensive hospital referrals due to acute respiratory system involvement, along with other symptoms. Although patients were not treated with any particular drug or vaccine, the physicians faced moral and legal aspects of the disease. Under such circumstances, the responsibility of making decisions about home quarantine, patient discharge, calling healthcare workers back to work after recovery from infection, etc., lied on the shoulders of first-line physicians (5-8) . The extensive patient load of emergency departments (EDs), as well as the lack of adequate knowledge of the virus behavior on a global scale, were other problems these physicians faced. During the COVID-19 pandemic, most of the ethical principles in medicine such as patient’s rights, became challenging. The current protocols do not clearly indicate how to deal with a non-cooperative patient; this can become legally and ethically challenging for physicians (9-11). Therefore, the current study aimed at evaluating these ethical and legal issues by reporting some cases.

The current case series study reports 10 patients who filed complaints against medical staff for problems that occurred on arrival, during the hospital stay or discharge in Shohada-ye-Tajrish and Shahid Modarres educational Hospitals, Tehran, Iran, from 20.02.2020 to 19.04.2020. Consultation with forensic medicine department was requested for all patients and final decisions for each case was reported. Patients with suspected COVID-19 infection were excluded, and there were no gender or age limitations in the study. All the data collected from patients remained confidential. The study protocol was approved by the Ethics committee of Shahid Beheshti University of Medical Sciences (Research plan tracking code is IR.SBMU.RETECH.REC.1399.164).

## Case presentation

**Case1:**

The patient was a 51-year-old overweight female with critical conditions complaining of dyspnea, cough, and myalgia, vertigo, taste disorder, and hyposmia. She was admitted to the emergency department (ED) for further examinations. Her vital signs were: Temperature (T) = 38/5°^.^C, blood pressure (BP) = 120/80 mmHg, pulse rate (PR) = 104/min, respiratory rate (RR) = 24/min, O_2_ saturation (SPO_2_) = 93% in room air, and blood sugar (BS) = 112 mg/dL. After performing chest CT scan and other laboratory tests, her infection with COVID-19 was confirmed. Despite the physician's advice, she refused to stay in the hospital due to the challenge of the family.

**Legal considerations:**

In the examination of the patient from legal aspects, she was alert and oriented. She was married and had a 14-year-old child; her level of education was a high school diploma. She was well oriented, could answer the questions consciously, and was capable of taking care of herself. She had no problems with home-based self-quarantine, and her physician believed that she would be okay at home, and there was no need for the hospital stay since she was not in a critical condition. Therefore, despite her infection with a lethal virus, there was no action to prohibit her from discharge; hence, the patient was discharged with prescription and advice about alarming signs and revisiting after two weeks of home quarantine.

**Case2:**

The patient was a 63-year-old male with obesity, cold symptoms, and loss of taste and smell senses, which had started five days before the visit. He was admitted to the ED, and his vital signs were: T = 39.5^°^C, BP = 105/70 mmHg, PR = 104/min, RR = 24/min, SPO_2_ = 90% in room air, and BS = 122 mg/dL. He also had heroin addiction. He was diagnosed with COVID-19 infection based on PCR results. He had withdrawal signs and respiratory distress due to infection in the lower respiratory tract.

**Legal considerations:**

The patient had been a heroin addict for about 15 years. He had a high school diploma and was not willing to stay in hospital due to addiction; but his discharge could be a hazard to society, due to infection with COVID-19. Therefore, due to his unstable conditions and in order to prevent worsening of the COVID-19 outbreak, his admission to intensive care unit (ICU) was recommended. He received IV medications for addiction withdrawal in addition to routine COVID-19 treatments. His discharge was prohibited, and since he could not make his own medical decisions, the healthcare system informed the hospital authorities.

**Case3:**

A 57-year-old male patient with obesity and type 2 diabetes complaining of myalgia and shortness of breath, taste disorder, and vertigo, referred to the hospital. His vital signs were: T = 39^°^C, BP = 100/80 mmHg, PR = 114/min, RR = 27/min, SPO_2_ = 88% in room air, and BS = 222 mg/dL. He was admitted to the general ward but then transferred to the ICU due to infection with COVID-19. His family insisted on transferring him to a private hospital.

**Legal considerations**:

The patient was not conscious and could not make his own medical decisions. Due to patient overload in the ED, his eldest son decided to transfer him to a private hospital. Finally, the supervisor of the hospital accepted the patient’s transfer, while he should make sure that all the hygienic protocols were observed and the patient was safely admitted to that hospital. If a patient cannot make his/her medical decisions, the family members attending the center can make the decision. Written consent was obtained from them immediately.

**Case4:**

A 66-year-old male physician with obesity, who was constantly in contact with infected patients and had a history of recto-sigmoid tumor and chemotherapy was diagnosed with COVID-19 infection. His vital signs were: T = 38^°^C, BP = 130/80 mmHg, PR = 114/min, RR = 27/min, SPO_2_ = 96%, and BS = 99 mg/dL. He insisted on staying at home for treatment completion.

**Legal considerations:**

Considering the patient’s occupation and his familiarity with medical procedures and protocols of quarantine, he was discharged for a home-based self-quarantine for 14 days. He was warned to get back to the hospital if any of the symptoms worsened.

**Case5:**

The patient was a 38-year-old female in her 36^th^ week of gestation admitted to the ED with complaints of typical COVID-19 symptoms (fever, myalgia, shortness of breath) as well as olfactory and taste disorders. Her vital signs were: T = 38.5^ °^C, BP = 100/70 mmHg, PR = 100/min, RR = 27/min, SPO_2_ = 90%, BS = 99 mg/dL, and had no history of abortions or any other maternity problems. After routine examinations and laboratory tests, she was diagnosed with COVID-19 infection, but her husband wanted to take her home. 

**Legal considerations:**

Examinations revealed that she was well-oriented and capable of making her own medical decisions. According to the current laws, her husband could not discharge her despite her will. The only rights that husband and wife have toward each other are the ones indicated in the marriage contract. In addition, the fetus’s health was engaged with that of the mother; therefore, the patient could not be discharged. 

**Case6:**

A 40-year-old nurse who had previous contact with patients infected with COVID-19 and was obese but had no history of any particular disease was admitted to ED; her vital signs were: T = 39.5°C, BP = 125/70 mmHg, PR = 85/min, RR = 20/min, SPO2 = 96%, and BS = 88mg/dL. She had a high fever and shortness of breath, hyposmia, taste disorder and her chest CT scan showed a mild bilateral pleural effusion with haziness in the lower respiratory tract. She did not want to complete her treatment in the hospital since she had a young child. 

**Legal considerations:**

Although she was a member of the healthcare staff, due to the worsening of her health status, she could not be discharged despite her own will. She was not capable of taking care of herself, her child, and other family members, and did not have adequate facilities for home-quarantine. 

**Case7:**

The patient was a 47-year-old male with obesity admitted to ICU due to infection with COVID-19. His vital signs on admission were: T = 39.5^°^C, BP = 95/70 mmHg, PR = 144/min, RR = 30/min, SPO_2 = _80%, and BS = 86 mg/dL. Unfortunately, he expired after five days. His family wished to get his corpse and hold a funeral. 

**Legal considerations:**

This patient passed away due to infection with the new coronavirus; therefore, the cause of death had to be written clearly and legibly in the death certificate, and the corpse had to be transferred to the cemetery under restricted terms by special ambulance. Therefore, the corpse was not handed to the family.

**Case8:**

The patient was a 39-year-old male; he was overweight and had severe cold-like symptoms similar to those of COVID-19 as well as taste disorder and had drunk handmade alcohol to ease his problem. Unfortunately, he arrived at the hospital too late due to loss of consciousness, snowstorm in both eyes, and respiratory distress. His vital signs were: T = 36.5^°^C, BP = 95/70 mmHg, PR = 67/min, RR = 34/min, SPO_2 _=78%, and BS = 182 mg/dL. Medical evaluations revealed severe metabolic acidosis with serum methanol and ethanol levels of 60 and 140 mg/dL, respectively. He was also diagnosed with COVID-19 infection based on PCR results. Despite all the interventions, including hemodialysis (due to methanol intoxication), twice, he expired after 36 hours. 

**Legal considerations:**

Considering the prohibition of alcohol consumption in Iran and the fact that the patient had consumed alcohol to treat his disease due to existing rumors, it was the responsibility of the Attorney General of Tehran to confront those spreading these rumors. His death certificate could be issued without an autopsy, since the cause of his death was methanol intoxication.

**Case9:**

The patient was a 61-year-old female with obesity and no history of drug abuse or disease. She had flu-like symptoms, olfactory disorder and considering the pandemic of COVID-19, her friends advised her to inhale opium to ease her conditions. She arrived at the hospital too late following respiratory distress. Her vital signs were: T = 37^°^C, BP = 100/80, PR = 65/min, RR = 34/min, SPO_2 _= 87%, and BS = 77 mg/dL; her PCR result for COVID-19 was positive. In addition, her morphine test result was positive, but she claimed that she had no idea about using drugs.

**Legal considerations:**

The patient did not want her parents to be informed about drug abuse. Therefore, since she was an adult and no consent was required, her drug abuse could not be elucidated, and she was treated for COVID-19 infection and the adverse effects of the drug. 

**Case10:**

The patient was a 48-year-old overweight female, who had a history of asthma for 15 years and took corticosteroids. She had symptoms of COVID-19 infection including fever and shortness of breath, as well as hyposmia but normal vital signs. Her chest x-ray showed haziness in the lower part of the right lung lobe, but the chest CT scan image showed healthy lungs. Her COVID-19 IgM test and PCR results were negative, but the COVID-19 IgG test was positive. The first-year general practice resident refused to discharge her due to suspicion of infection. 

**Legal considerations:**

After more evaluations from a legal point of view and studying her blood gas analysis, it seemed that her asthma was poorly controlled during the past months. She was discharged after consultation with an infectious disease specialist since her infection with COVID-19 was improbable. 

Characteristics of reported cases are summarized in table 1.

**Table1:** Characteristics of reported cases

**Cases**	**Gender**	**Age**	**Condition**	**PCR **	**IgM **	**IgG **	**Chest CT Scan**
C1	Female	51	Ill	Pos^+^	Pos^+^	Neg^-^	Not significant
C2	Male	63	Not good	Pos^+^	Pos^+^	Pos^+^	Haziness in the right lobe
C3	Male	57	Ill	Pos^+^	Pos^+^	Pos^+^	Haziness in the right and left lobes
C4	Male	66	Fair	Pos^+^	Pos^+^	Neg^-^	Normal
C5	Female	38	Ill	Pos^+^	Neg^-^	Neg^-^	Normal
C6	Female	40	Bad	Neg^-^	Pos^+^	Neg^-^	Tiff in the right lobe
C7	Male	47	Bad	Pos^+^	Pos^+^	Pos^+^	Haziness in the right and left lobes
C8	Male	39	Ill	Pos^+^	Pos^+^	Neg^-^	Not done
C9	Female	61	Bad	Neg^_^	Pos^+^	Pos^+^	Haziness in the right lobe
C10	Female	48	Good	Neg^-^	Neg^-^	Pos^+^	Normal

PCR: polymerase chain reaction; CT: computed tomography.

## Discussion

According to the legal and canonical sources in Iran, there are six groups of interdicted people, who cannot be in charge of actions: 1) People with fatal diseases, 2) children, 3) mentally-ill people, 4) insane people, 5) slaves, and 6) real bankrupts (12-14). People infected with COVID-19 are assigned to the first group, which includes all the patients admitted to ED, ICU, critical care unit (CCU), etc., as such patients are infected with a fatal disease and are also a major risk for the health of society. Consequently, since they are not in the condition to think for themselves, the healthcare system should make medical decisions for them (15-18). 

As mentioned in the article by Cristina Cattaneo, COVID-19 has affected the practice of forensic medicine. In the previous years, the focus had been on preparing protocols for recovery and identification of victims or collecting evidence and recreating the victims’ manner of death in disasters such as explosions, tsunamis, and mass disasters, or homicide, child abuse, or manslaughter, which are very different from a virus pandemic in nature. The pandemic has not only forced a reduction in medicolegal autopsies it has also increased the number of living victims in need of medicolegal services. While in some cases the victims cannot be assisted due to lockdown or restrictions (19). 

We are faced with global economic and social issues in addition to our healthcare system challenges. Mandatory quarantine was an approach used in many countries, but it has its own drawbacks too. For example, governments must ensure that people’s basic needs are met and that they have access to healthcare, medication, food, and sanitation. This is critical to ensure that they will comply with orders (20).

In the article by Terry Skolnik (21) it is stated that this global pandemic has provided an opportunity for judges, policy makers and justice system actors to make lasting positive changes to the system, similar to those observed during the pandemic. 

We could not find any similar studies as in most studies the issue has been addressed only from a diagnostic and therapeutic point of view and there is no mention of legal issues at all. In the event of any legal issues for patients, we suggest consulting with forensic experts.

## Conclusion:

It is necessary for the healthcare system to update itself based on the latest guidelines and interventions for COVID-19 and facilitate legal assistance in order to prevent future prosecutions.
